# Screening for undiagnosed hyperglycemia in patients with periodontitis: a cross-sectional study

**DOI:** 10.31744/einstein_journal/2026AO2397

**Published:** 2026-07-29

**Authors:** Fernanda Neves Amarante Gouveia, Paola Elizabete Bezerra da Silva Galvão, Aline Roberta Oliveira da Silva, Manoela Almeida Santos da Figueira, Júlia Bello Junqueira Ribeiro, Rafael Scaf de Molon, Mariana Fampa Fogacci, Davi da Silva Barbirato

**Affiliations:** 1 Faculdade Pernambucana de Saúde Dental School Recife PE Brazil Dental School, Faculdade Pernambucana de Saúde, Recife, PE, Brazil.; 2 Universidade Federal de Pernambuco Department of Clinical and Preventive Dentistry Recife PE Brazil Department of Clinical and Preventive Dentistry, Universidade Federal de Pernambuco, Recife, PE, Brazil.; 3 Instituto de Medicina Integral Professor Fernando Figueira Recife PE Brazil Instituto de Medicina Integral Professor Fernando Figueira, Recife, PE, Brazil.; 4 Universidade Federal de Minas Gerais Department of Public Health Belo Horizonte MG Brazil Department of Public Health, Universidade Federal de Minas Gerais, Belo Horizonte, MG, Brazil.; 5 Universidade Estadual Paulista Faculdade de Odontologia Department of Diagnostic and Surgery Aracatuba SP Brazil Department of Diagnostic and Surgery, Faculdade de Odontologia, Universidade Estadual Paulista, Aracatuba, SP, Brazil.; 6 Universidade de São Paulo Faculdade de Odontologia de Ribeirão Preto Department of Oral & Maxillofacial Surgery and Periodontology Ribeirao Preto SP Brazil Department of Oral & Maxillofacial Surgery and Periodontology, Faculdade de Odontologia de Ribeirão Preto, Universidade de São Paulo, Ribeirao Preto, SP, Brazil.

**Keywords:** Periodontal diseases, Periodontitis, Diabetes mellitus, Glycated hemoglobin, Diagnosis, Risk assessment, Hyperglycemia, Surveys and questionnaires

## Abstract

**Objective:**

We evaluated the potential of the Finnish Diabetes Risk Score questionnaire to screen for undiagnosed hyperglycemia in patients with periodontitis, comparing its performance with that of glycated hemoglobin (HbA1c).

**Methods:**

In this cross-sectional pilot study, we included 21 adults (22-64 years) diagnosed with periodontitis. Demographic data, periodontal status, HbA1c levels, and Finnish Diabetes Risk Scores were collected. Based on HbA1c levels, participants were classified as normoglycemic (<5.7%), prediabetic (5.7-6.4%), or diabetic (≥6.5%) according to established diagnostic criteria. Finnish Diabetes Risk Scores ≥12 indicated moderate-to-very-high diabetes risk. Associations between the Finnish Diabetes Risk Score, HbA1c, and periodontal parameters were assessed using Friedman's test and multinomial logistic regression. Diagnostic validity indices and receiver operating characteristic (ROC) curves were calculated.

**Results:**

Most participants were women (71.4%) and had advanced disease (71.4% stage IV; 71.4% generalized periodontitis). According to the Finnish Diabetes Risk Score, 71.5% of patients had a high or very high risk of developing type 2 diabetes within 10 years. Mean HbA1c was 5.8±0.4%, ranging from normoglycemic to diabetic levels (≥6.5%). Finnish Diabetes Risk Scores ≥12 were significantly associated with HbA1c-defined prediabetes/diabetes and generalized periodontitis (p<0.001). In the multinomial logistic regression analysis, age, sex, periodontal extent, and HbA1c were identified as independent predictors of higher Finnish Diabetes Risk Scores. Finnish Diabetes Risk Scores ≥12 showed high sensitivity (92-100%) and negative predictive value (80-100%), but low specificity (26-44%); ROC analysis demonstrated acceptable diagnostic performance, particularly for identifying combined prediabetes and diabetes.

**Conclusion:**

The Finnish Diabetes Risk Score is a practical, non-invasive screening tool for predicting the risk of undiagnosed dysglycemia in patients with periodontitis in dental settings. Its high sensitivity supports its use for risk stratification; however, positive findings require biochemical confirmation. These findings underscore the role of dental professionals in early disease detection.

## Highlights

Finnish Diabetes Risk Score identified high diabetes risk in most patients with periodontitis.

High or very high T2DM risk was observed in 71.5% of participants.

Finnish Diabetes Risk Score ≥12 was associated with dysglycemia and severe periodontitis.

Dental clinics may help detect undiagnosed hyperglycemia early.

## In Brief

Patients with periodontitis frequently presented with elevated diabetes risk and undiagnosed dysglycemia. Finnish Diabetes Risk Score demonstrated high sensitivity for identifying hyperglycemia risk and may serve as a practical, non-invasive screening tool in dental settings, supporting early detection and referral for medical evaluation.

## Graphical abstract

**Figure:**
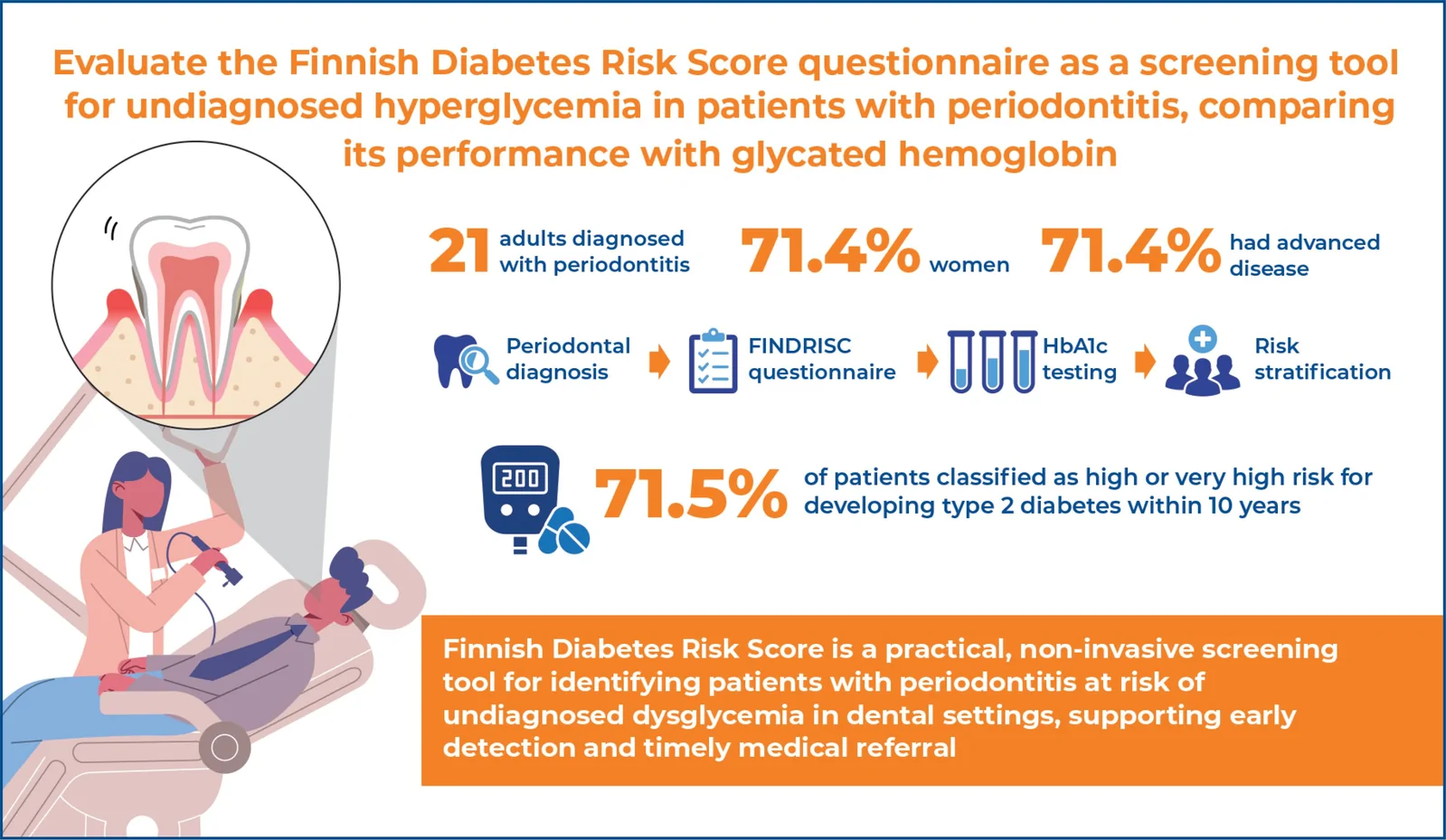

## INTRODUCTION

Periodontitis is a prevalent, multifactorial, chronic inflammatory disease associated with a dysbiotic biofilm and characterized by the progressive destruction of tooth-supporting periodontal tissues.^(1–4)^ It results from a complex interaction between pathogenic microorganisms and the host immune-inflammatory response, which, when dysregulated, leads to tissue breakdown and alveolar bone loss.^(5–9)^ Although the disease may persist over time, it can be effectively controlled using mechanical debridement aimed at disrupting and removing the subgingival biofilm, performed either alone or in combination with adjunctive therapies such as antimicrobial agents, host-modulatory approaches, or other supportive interventions, followed by appropriate maintenance care.^(10–13)^ Moreover, the systemic implications of this chronic inflammatory burden have become evident, as periodontitis does not only compromise oral health but also interacts with various systemic diseases.^(14–17)^

One of the most relevant systemic associations of periodontitis is with *diabetes mellitus* (DM).^(14,18,19)^ *Diabetes mellitus* is a widespread metabolic disease caused by impaired insulin secretion, resistance, or a combination of both.^(18)^ Approximately 90% of individuals with type 2 *diabetes mellitus* (T2DM).^(18, 20)^ The global burden of diabetes has risen dramatically from 108 million in 1980 to 422 million in 2014. Current projections suggest this figure will more than double in the coming 20 years, making diabetes one of the most pressing public health concerns worldwide. The World Health Organization estimates that by 2030, diabetes will rank as the seventh leading cause of death globally.^(21)^

Current evidence demonstrates a bidirectional relationship between T2DM and periodontitis.^(19,22,23)^ *Diabetes mellitus* predisposes individuals to periodontitis and fosters its progression,^(24,25)^ and periodontitis worsens glycemic control and is the sixth most significant complication of DM.^(24)^ The biological mechanisms of this relationship involves chronic hyperglycemia, which promotes the formation and accumulation of advanced glycation end-products in plasma and tissues, thereby intensifying the inflammatory response within periodontal tissues. Patients with T2DM and severe periodontitis experience a greater decline in glycemic control over time compared with those without.^(26)^ These findings suggest that periodontitis can precede and contribute to worsening metabolic control, reinforcing the need for integrated management of both conditions.^(27)^

A major challenge in the management of T2DM is its underdiagnosis. The disease often develops insidiously, with mild or absent symptoms in its early stages, leading to delayed recognition and intervention.^(28,29)^ The consequences of this delayed diagnosis are profound, as untreated T2DM significantly increases the risk of chronic complications, including periodontitis. Data from the Brazilian Diabetes Society indicate that approximately 20 million Brazilians currently live with T2DM, half of whom are unaware of their condition. This silent burden of undiagnosed diabetes imposes severe biopsychosocial consequences, reduces quality of life and life expectancy, and contributes to escalating healthcare costs.^(28)^

Fasting blood glucose and glycated hemoglobin (HbA1c) are the gold standards for identifying and monitoring T2DM. HbA1c is used to confirm the presence of dysglycemia and to validate the bidirectional link between periodontitis and diabetes.^(28, 29)^ However, these tests require laboratory infrastructure, which may not be readily accessible in underserved populations or certain healthcare systems. This limitation highlights the need for feasible, low-cost, and non-invasive screening strategies that can complement traditional diagnostic approaches and allow for earlier identification of at-risk individuals.^(30–33)^

Dental care providers, especially periodontists, play a major role in this context. Since dental visits are an opportunity to systemically assess patients, dentists are uniquely positioned to identify individuals at risk for diabetes. Many cases of prediabetes and DM are first suspected during dental appointments, particularly when periodontitis is diagnosed. Incorporating validated risk assessment tools into dental practice may therefore be an important step in bridging the gap between oral health care and systemic disease prevention. The Finnish Diabetes Risk Score (FINDRISC) questionnaire is a simple, validated, and widely used instrument for estimating the 10-year risk of developing T2DM.^(34)^ Its application in dental settings, combined with clinical periodontal diagnosis, could represent a practical and non-invasive approach for screening undiagnosed hyperglycemia and identifying patients at high risk of diabetes.

Thus, we aimed to evaluate the relationship between clinical periodontal diagnosis, HbA1c levels, and the risk of developing T2DM in the next 10 years using the FINDRISC questionnaire ^(34)^ among patients with periodontitis treated at a dental school clinic. By exploring this association, we sought to address two critical gaps: (i) the lack of accessible screening methods for early identification of diabetes risk in dental settings, and (ii) the underexplored potential of dental professionals in contributing to the prevention and early detection of systemic diseases.

## OBJECTIVE

We evaluated the potential of the Finnish Diabetes Risk Score questionnaire to screen for undiagnosed hyperglycemia in patients with periodontitis, comparing its performance with that of glycated hemoglobin (HbA1c).

## METHODS

### Study design

This was a comparative observational pilot study with a cross-sectional and analytical design, conducted among patients undergoing periodontal diagnosis, recruited using the convenience sampling technique. The primary objective was to investigate the potential of the FINDRISC questionnaire for screening T2DM among adult patients diagnosed with periodontitis. The FINDRISC score; demographic and clinical variables including sex, age, HbA1c; and the presence of prediabetes were assessed.

The study protocol was reviewed and approved by the Research Ethics Committee of the *Associação Educacional de Ciências da Saúde* (CAAE: 87504425.0.0000.5569; # 7.588.647). Patients consented to participation and publication of their data by signing the Free and Informed Consent Form in accordance with the Brazilian National Health Council Resolution CNS No. 466/2012. All procedures complied with the principles of the Declaration of Helsinki (1964) and its seven subsequent revisions, with the most recent update in 2013. This study adhered to the *Strengthening the Reporting of Observational Studies in Epidemiology* guidelines to ensure methodological rigor and transparency.^(35)^ We hypothesized that FINDRISC could serve as a useful tool for the early identification of undiagnosed diabetes or prediabetes in individuals affected by periodontitis.

### Participants

We included patients undergoing routine periodontal examinations at the Odonto FPS Clinic of the *Faculdade Pernambucana de Saúde* (FPS). Individuals were eligible if they were adults aged 22-64 years and diagnosed with periodontitis. We excluded patients with a prior diagnosis of prediabetes or DM (type 1 or type 2), those who had undergone periodontal treatment within the previous 6 months, and individuals who had used anti-inflammatory or antibiotic medications during the preceding 3 months. Patient recruitment was performed by one of the researchers, who applied the inclusion and exclusion criteria consistently to ensure sample homogeneity. Overall, 21 patients were included in the analysis.

### Examination protocol

Relevant demographic and clinical information was obtained from the participants’ medical records, including sex, age, periodontal diagnosis, and HbA1c levels. The periodontal clinical diagnosis was established in accordance with the most recent classification system for periodontal and peri-implant diseases, jointly published by the American Academy of Periodontology (AAP) and the European Federation of Periodontology (EFP).^(36)^ Blood glucose status was determined through HbA1c measurement, and participants were categorized as normoglycemic (HbA1c <5.7%), prediabetic (HbA1c 5.7-6.4%, 39-47 mmol/mol), or diabetic (HbA1c ≥6.5%, ≥48 mmol/mol), following internationally recognized diagnostic thresholds.^(37–40)^ These variables were recorded as both continuous quantitative data and categorical qualitative outcomes. Individuals presenting with HbA1c values ≥5.7% were informed of their results and advised to consult their physician for further medical evaluation and diagnosis. The findings were explained directly to the participants, who were encouraged to share them with their healthcare provider.

Each participant completed the self-administered FINDRISC^(34)^ questionnaire under the supervision of the researcher. The FINDRISC questionnaire is a validated, non-invasive screening tool designed to estimate the 10-year risk of developing T2DM.^(34)^ It consists of eight items assessing key risk factors, including age, body mass index (BMI), waist circumference, level of physical activity, dietary habits (particularly fruit and vegetable consumption), history of antihypertensive medication use, prior episodes of elevated blood glucose, and family history of diabetes. Each item is assigned a weighted score, and the total score ranges from 0 to 26 points, with higher values indicating greater risk. Based on the cumulative score, individuals were categorized into risk groups ranging from low to very high probability of developing diabetes within the next 10 years.

### Outcomes

The primary outcome was the correlation between the FINDRISC score and HbA1c status, with specific interest in identifying patients at moderate to very high risk of developing T2DM within the next decade.^(34)^ Consistent with the study by Monteiro et al.^(41)^ a FINDRISC score ≥12 was considered indicative of moderate to very high risk in our study and was correlated with HbA1c values suggestive of prediabetes or diabetes. In the analysis, FINDRISC was treated as both a discrete quantitative variable and a categorical variable stratified into low, slightly elevated, moderate, high, and very high-risk groups.^(34)^ Participants were classified as potentially T2DM-positive (FINDRISC ≥12) or not (FINDRISC <12).

Secondary outcomes addressed the diagnostic validity of the FINDRISC questionnaire compared to HbA1c-based diagnosis. To this end, diagnostic accuracy measures were calculated by constructing 2×2 contingency tables to classify participants as true positives, true negatives, false positives, or false negatives relative to HbA1c thresholds. From these classifications, standard diagnostic indices were computed, including sensitivity, specificity, positive predictive value (PPV), and negative predictive value (NPV). Likelihood ratios (LRs) were also derived: the positive LR (LR+) quantified the increased probability of disease given a positive test, while the negative LR (LR-) reflected the probability of disease in the presence of a negative result. Accuracy was calculated as the proportion of correctly classified cases across all test results. Receiver operating characteristic (ROC) curve analysis was performed to visualize the trade-off between sensitivity and specificity across thresholds. The area under the ROC curve (AUC) was used as a global indicator of diagnostic performance, with values between 0.7 and 0.8 indicating acceptable accuracy, 0.8 to 0.9 reflecting good to excellent performance, and values above 0.9 considered outstanding.

### Statistical analysis

All statistical analyses were conducted using the Statistical Package for the Social Sciences (SPSS® version XX, IBM Corporation, Armonk, NY, USA). Descriptive statistics were applied to summarize categorical variables as absolute frequencies and percentages, and numerical variables as mean±standard deviation. Variables including sex, age, and FINDRISC score ≥12 were analyzed as dichotomous categories (male vs. female; adults (20-59 years) vs. older adult (≥60 years); and screening positive (≥1) *vs.* negative (<12). Glycemic status was classified as normoglycemia, prediabetes, and diabetes. Periodontitis was staged and graded according to the AAP/EFP system, and risk of developing T2DM based on FINDRISC was categorized from low to very high. HbA1c and FINDRISC scores were also retained as continuous variables for quantitative analysis.

Pearson's test χ^2^ was used to examine associations between categorical variables, and Skewness and Kurtosis tests were used to assess data normality. The Friedman's test was used to compare numeric and nominal variables. A multinomial logistic regression model was employed to predict non-ranked categorical outcomes with more than two levels. The diagnostic validity of the FINDRISC questionnaire (≥12 *vs.* <12) was analyzed as described above. Statistical significance was set at p≤0.05 for all tests.

## RESULTS

Overall, 21 patients with periodontitis were included in the study. Most participants were women (71.4%) and presented with stage IV, grade B, generalized periodontitis,. Regarding the distribution pattern, more than 30% of the dentition was affected in 71.4% of participants (Table 1).

**Table 1:** Descriptive data

Variables		Frequency	Valid percent	Histogram
Sex				
	Male	6	28.6	
	Female	15	71.4	
Age, years				
	Adults (20-59	10	47.6	
	Older adult (≥60)	11	52.4	
Periodontitis				
	Stage I	3	14.3	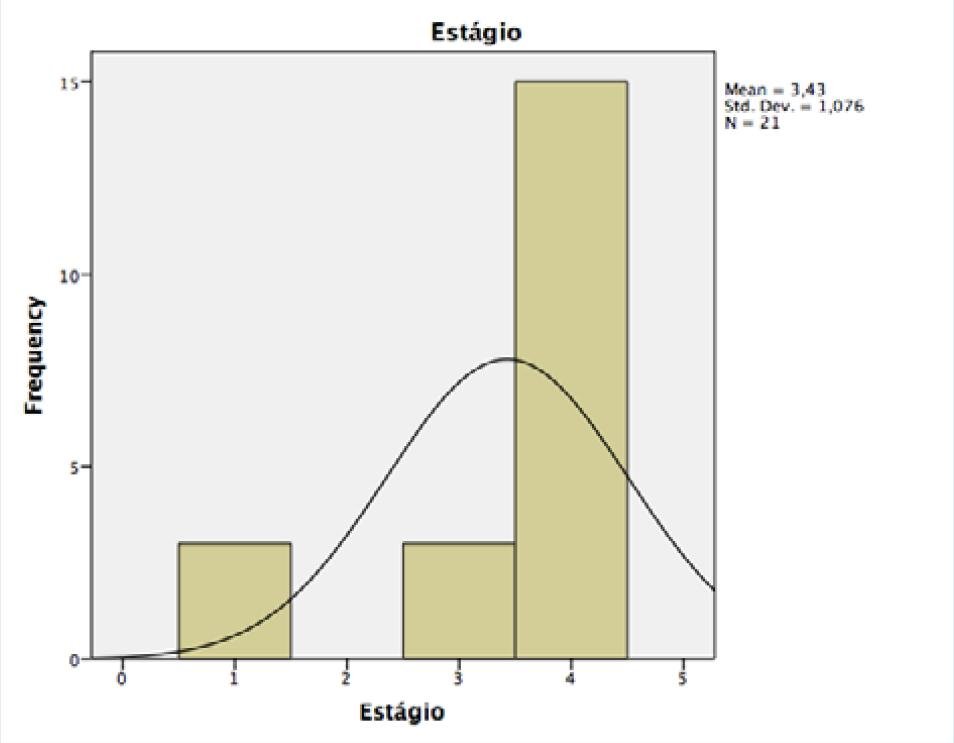
	Stage III	3	14.3	
	Stage IV	15	71.4	
	Localized	6	28.6	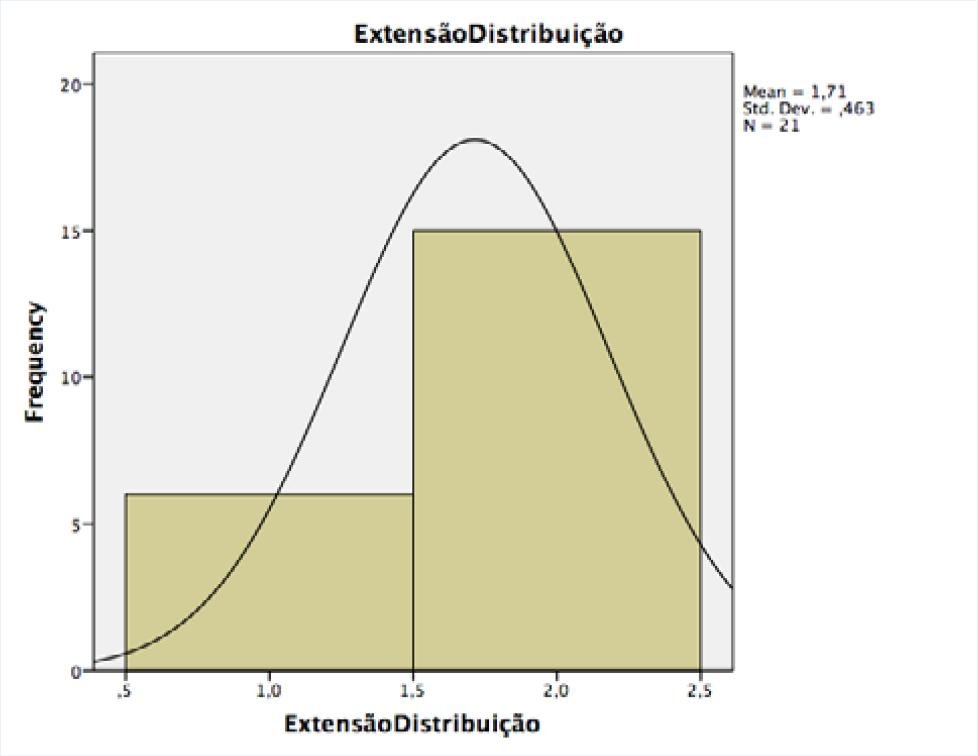
	Generalized	15	71.4	
	Grade A	2	9.5	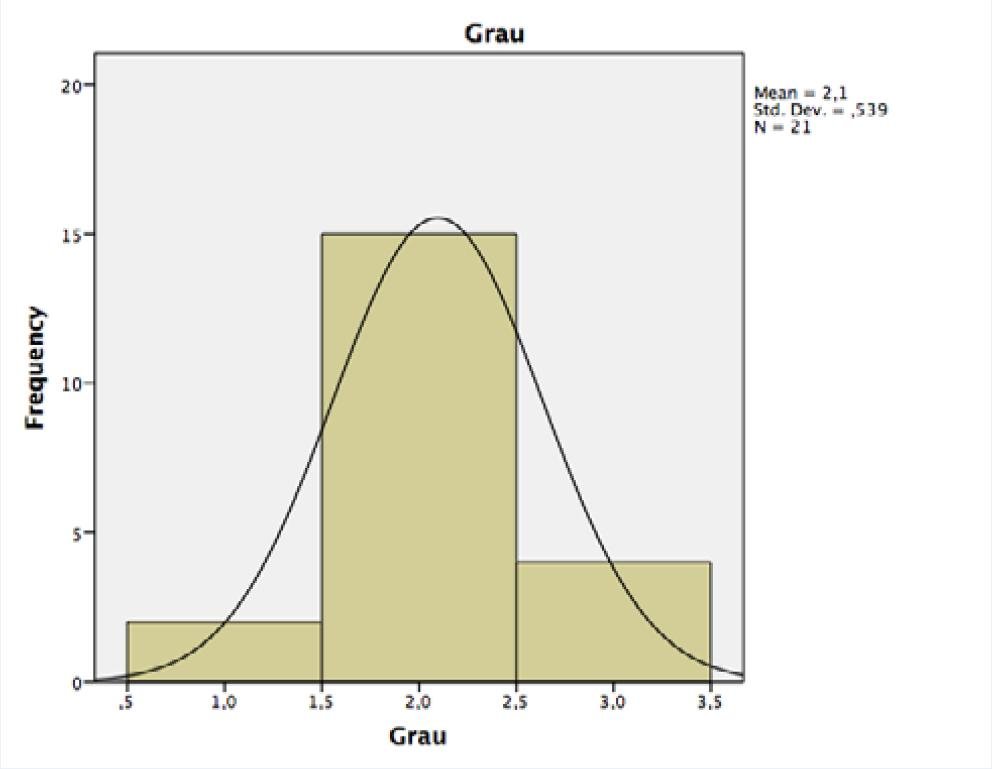
	Grade B	15	71.4	
	Grade C	4	19	
FINDRISC score				
	Low (1:100)	2	9.5	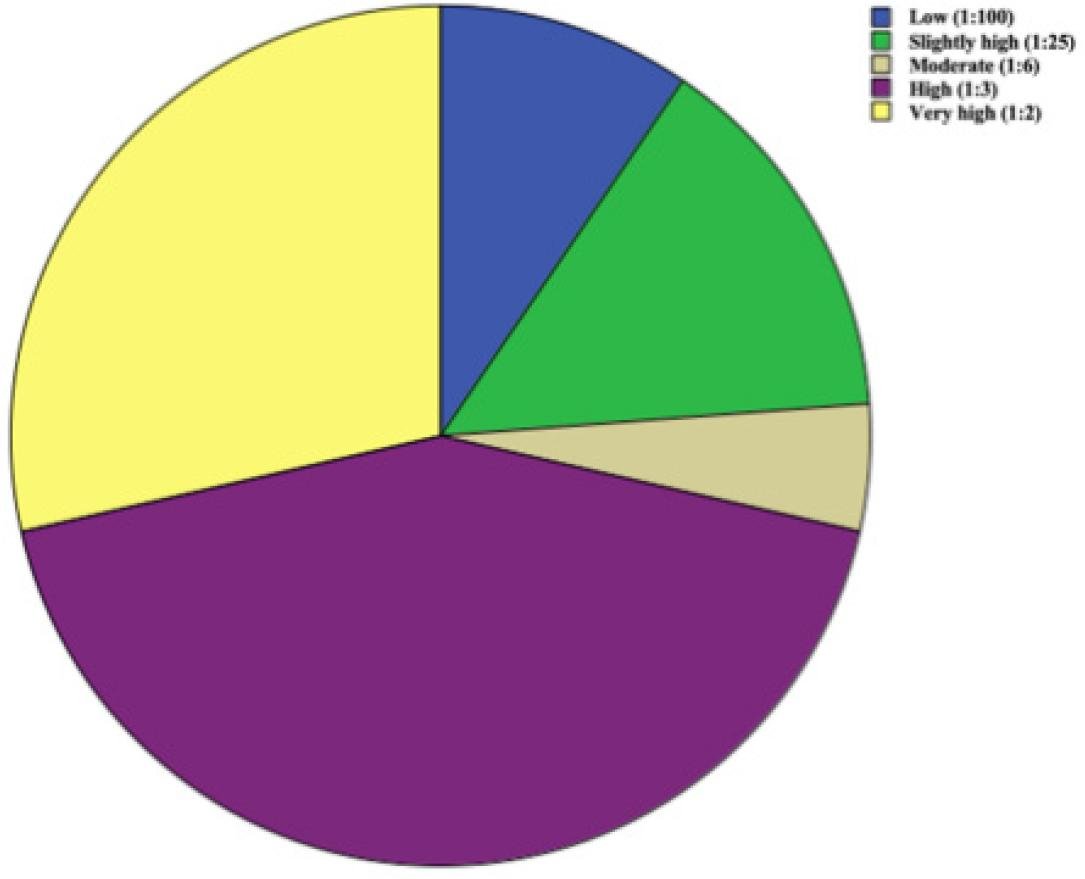
	Slightly high (1:25)	3	14.3	
	Moderate (1:6)	1	4.8	
	High (1:3)	9	42.9	
	Very high (1:2)	6	28.6	

FINDRISC: Finnish Diabetes Risk Score.

Regarding age distribution, 47.6% of patients were classified as adults (20-59 years), and 52.4% as older adults (≥60 years). In terms of diabetes risk, 71.5% of the participants had either a high (42.9%) or very high (28.6%) probability of developing T2DM within the next 10 years, as estimated by the FINDRISC questionnaire. Only a small proportion of patients had low (9.5%), slightly elevated (14.3%), or moderate risks (4.8%) (Table 1).

The mean HbA1c value across participants was 5.8±0.4%, ranging from 5.5% to 7%. Although the mean HbA1c values were within the prediabetic range, some participants were already at or above the diagnostic threshold for T2DM (≥6.5%). The distribution of HbA1c data was asymmetric, reflecting variability in glycemic control within the sample. Similarly, FINDRISC scores ranged widely, from 4 to 24 points, with a mean of 16±5.6 points, demonstrating skewed data distribution (Table 2).

**Table 2:** Descriptive statistics of HbA1c and FINDRISC measurements

Variable	N	Min.	Max.	Mean	Sth. Deviation	Skewness		Kurtosis	
						Statistic	Sth. Error	Statistic	Sth. Error
HbA1c	21	5.5	7	5.8	0.4	2.233	0.501	5.646	0.972
FINDRISC	21	4	24	16	5.6	-0.697	0.501	0.221	0.972

FINDRISC: Finnish Diabetes Risk Score; HbA1c: Glycated hemoglobin; Std: Standard.

A FINDRISC score ≥12 was significantly associated with both HbA1c-defined prediabetes/diabetes status and the extent of periodontal involvement. Patients with higher FINDRISC scores were more likely to exhibit generalized periodontitis and HbA1c values consistent with prediabetes or diabetes (p<0.001, Friedman test) (Table 3).

**Table 3:** Correlation between FINDRISC score ≥12 and HbA1c-prediabetes/T2DM and extension/distribution of periodontitis stage

Ranks	Mean Rank	Test statistics*	
		n	21
FINDRISC score	1.90	χ^2^	15.211
Blood glucose	1.10	df	1
(Categories: HbA1c normal, prediabetes or T2DM)		Asymp. Sig.	0.000
	**Mean Rank**	**n**	**21**
FINDRISC score	1.88	χ^2^	12.800
Extension/Distribution of periodontitis stage	1.12	df	1
(Categories: localized, generalized or molar/incisor pattern)		Asymp. Sig.	0.000

* Friedman Test.

FINDRISC: Finnish Diabetes Risk Score; HbA1c: Glycated hemoglobin.

The multinomial logistic regression model, which was used to evaluate the influence of demographic and clinical factors on FINDRISC scores, was significant (p=0.027). Within this model, several predictor variables showed a strong and independent effect on the likelihood of obtaining a FINDRISC score ≥12 Including age, sex, the extent/distribution of periodontitis, and HbA1c values. Notably, older age, female sex, generalized periodontal involvement, and elevated HbA1c were strongly associated with higher FINDRISC scores. Contrarily, the stage and grade of periodontitis did not significantly predict FINDRISC outcomes (Table 4 and 5).

**Table 4:** Multinomial logistic regression model

Model	Model Fitting Information			
	Model Fitting Criteria	Likelihood Ratio Tests		
	-2 Log Likelihood	χ^2^	df	Sig.
Intercept Only	23.053			
Final	0.000	23.053	12	0.027

**Table 5:** Predictor variables significantly affecting FINDRISC score ≥12

Effect	Likelihood Ratio Tests			
	Model fitting criteria	Likelihood Ratio Tests		
	-2 Log Likelihood of reduced model	χ^2^	df	Sig.
Intercept	0.000*	0.000	0	.
Age	11.673	11.673	1	0.001
Sex	14.377	14.377	1	0.000
Stage	2.773#	2.773	1	0.096
Extent/Distribution	13.274	13.274	1	0.000
Grade	0.091#	0.091	1	0.763
HbA1c	13.907	13.907	5	0.016
Glycemia	0.000*	0.000	0	.

The χ^2^ statistic is the difference in −2 log-likelihoods between the final model and a reduced model. The reduced model was formed by omitting an effect from the final model. The null hypothesis is that all parameters of that effect are 0.

* This reduced model is equivalent to the final model because omitting the effect does not increase the degrees of freedom;

# Unexpected singularities in the Hessian matrix are encountered. This indicates that either some predictor variables should be excluded or some categories should be merged.

FINDRISC: Finnish Diabetes Risk Score; HbA1c: Glycated hemoglobin.

The diagnostic validity of the FINDRISC questionnaire was evaluated by comparing its performance against HbA1c classification. When considering T2DM alone (Model 1), a FINDRISC score ≥12 demonstrated perfect sensitivity (100%), correctly identifying all patients with HbA1c-confirmed diabetes. However, specificity was low (26%), as many participants without diabetes were incorrectly classified as positive. The PPV was 13%, indicating that only a small proportion of positive FINDRISC results corresponded to true diabetes cases. In contrast, the NPV was 100%, meaning that a negative FINDRISC score reliably excluded patients with diabetes (Table 6).

**Table 6:** Diagnostic validity of the FINDRISC questionnaire (FINDRISC score ≥12)

Test performance	Model 1	Model 2
	TP=T2DM | TN=HbA1c normal + prediabetes	TP=Prediabetes + T2DM | TN=HbA1c normal
Sensitivity TP/(TP+FN)	2/(2+0)=1	11/(11+1)=0.92
Specificity TN/(TN+FP)	5/(5+14)=0.26	4/(4+5)=0.44
Positive Predictive Value (PPV) TP/(TP+FP)	2/(2+14)=0.13	11/(11+5)=0.69
Negative Predictive Value (NPV) TN/(TN+FN)	5/(5+0)=1	4/(4+1)=0.8
Likelihood Ratios (LR)		
Positive LR Sensitivity / (1-Specificity)	1/(1-0.26)=1.35	0.92/(1-0.44)=1.64
Negative LR (1-Sensitivity) / Specificity	(1-1)/0.26=0	(1-0.92)/0.44=0.18
Accuracy (TP+TN)/(TP+TN+FP+FN)	(2+5)/21=0.33	(11+4)/21=0.71

TP: true positives; TN: true negatives; FP: false positives; FN: false negatives; T2DM: type 2 *diabetes mellitus*; HbA1c: Glycated hemoglobin.

When the definition of "disease" was broadened to include both prediabetes and diabetes (Model 2), sensitivity remained high (92%), and specificity improved slightly (44%). The PPV increased substantially to 69%, suggesting that patients with a positive FINDRISC result had a two-thirds chance of actually having prediabetes or diabetes. The NPV was 80%, indicating that a negative result continued to provide strong reassurance of normoglycemia. Overall accuracy improved from 33% in Model 1 to 71% in Model 2.

Likelihood ratio analysis further supported these conclusions. In Model 1, the LR+ was 1.35, suggesting limited utility in confirming diabetes, while the LR- was 0, reflecting excellent reliability in excluding the disease. In Model 2, the LR+ increased to 1.64, and the LR- was 0.18.

ROC curve analysis illustrated the trade-off between sensitivity and specificity at different thresholds. The area under the ROC curve (AUC) indicated reasonable to acceptable performance of the FINDRISC score ≥12 for screening prediabetes and diabetes (Figure 1).

**Figure 1:**
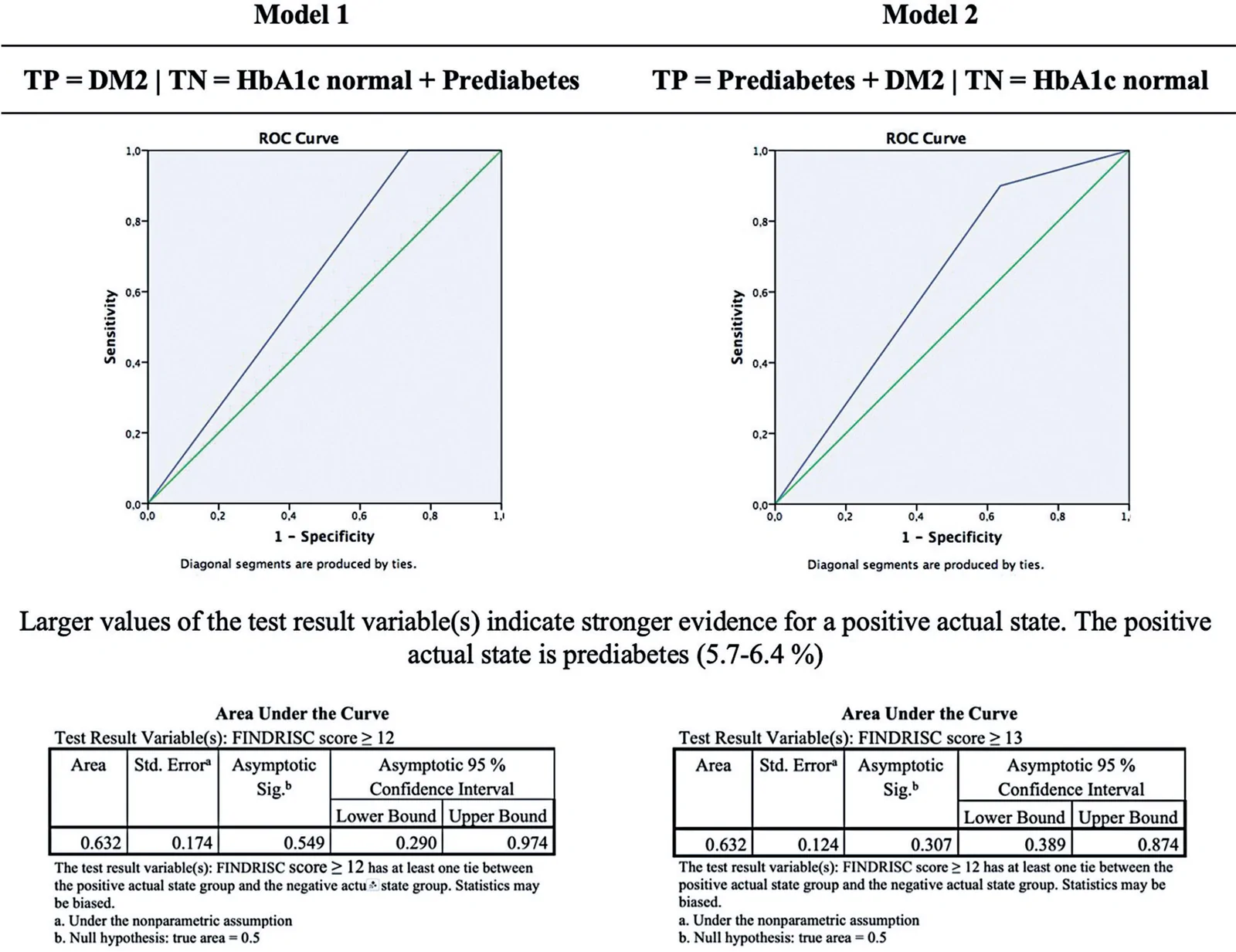
Performance of FINDRISC as a binary classifier (Model 1 and Model 2), showing the TP rate (sensitivity) *versus* the FP rate (specificity) for three diagnostic thresholds (normal HbA1c, prediabetes, and T2DM [categories of Glycemic variable])

TP: true positives; TN: true negatives; FP: false positives; FN: false negatives; T2DM: type 2 diabetes mellitus; HbA1c: Glycated hemoglobin; FINDRISC: Finnish Diabetes Risk Score.

## DISCUSSION

The results of this study demonstrate that FINDRISC is a sensitive, though not highly specific, tool for detecting undiagnosed hyperglycemia among patients with periodontitis. It accurately identifies patients with scores below the threshold of 12 as negative; however, positive results should be interpreted with caution and confirmed through laboratory testing. Importantly, the overlap between high FINDRISC scores, HbA1c-defined prediabetes/diabetes, and generalized periodontitis underscores the intertwined nature of metabolic and oral inflammatory diseases, reinforcing the need for integrated screening strategies in dental settings.

This tool is particularly important given the recommendations of the joint IDF-EFP workshop, which highlighted the central role of dental professionals in the early identification of hyperglycemia. Such a role becomes even more critical in the context of periodontitis, where HbA1c assessment and systematic screening for prediabetes and T2DM are strongly recommended. ^(36–40,42,43)^

The FINDRISC questionnaire is one of the most widely validated and applied screening tools for predicting the risk of developing T2DM.^(29,34,44)^ Its use is an important first step in identifying previously undiagnosed hyperglycemia.^(45)^ Several studies in diverse populations have already confirmed its diagnostic validity, with AUC ranging from 0.73 in Latin American cohorts to 0.63 in African populations.^(46,47)^

Recent evidence has highlighted the advantages of combining FINDRISC with HbA1c assessment. The DiabetRisk Study conducted by Montero et al.^(41)^ demonstrated that a protocol integrating FINDRISC, HbA1c measurement, and optionally a baseline periodontal examination was effective for identifying individuals with undiagnosed hyperglycemia across a large network of dental clinics. In alignment with these findings, our study reinforces the potential of a combined approach, especially in dental settings where access to biochemical testing may be limited.

According to current classification criteria, HbA1c levels are essential for grading periodontitis severity and establishing personalized treatment plans.^(36–40,42,43)^ Furthermore, the bidirectional relationship between periodontitis and T2DM^(19,48–53)^ supported the recommendation of screening for prediabetes and T2DM among all individuals with periodontitis. Nonetheless, in many clinical and epidemiological contexts, biochemical testing is not feasible due to infrastructure, cost, or access limitations. This practical barrier underscores the importance of validated non-invasive tools such as FINDRISC. By providing an initial, accessible means of identifying at-risk individuals, FINDRISC offers both clinical (patient-level) and epidemiological (population-level) relevance. Our study demonstrated that patients with a FINDRISC score ≥12, categorized as having moderate to very high risk, were significantly associated with HbA1c-defined prediabetes and T2DM. Importantly, the sensitivity of FINDRISC in this cohort exceeded 70%, which is considered an acceptable threshold for a screening tool according to Montero et al.^(41)^ This aligns with the diagnostic validity reported in previous studies and confirms the potential of FINDRISC to serve as a practical first-line screening instrument among patients with periodontitis.

Nevertheless, it is important to note that while FINDRISC ≥12 achieved high sensitivity, it showed relatively low specificity. In other words, the tool was effective in identifying true positives but generated a considerable number of false positives. This reinforces the recommendation that FINDRISC should not be used as a standalone diagnostic method but rather as an initial screening measure that requires confirmation through biochemical tests. Convenient alternatives to traditional laboratory-based HbA1c measurement, such as point-of-care devices, may help overcome logistical barriers while maintaining acceptable accuracy. ^(54)^

The factors influencing a FINDRISC score ≥12 in our population included sex, age, extent and distribution of periodontitis, and HbA1c-defined glycemic status. Specifically, female sex, older age, generalized periodontitis, and confirmed prediabetes or T2DM were significantly associated with higher FINDRISC scores. These findings not only align with the multifactorial nature of periodontitis but also highlight how systemic and local risk factors may converge to increase the probability of metabolic impairment. Importantly, early detection of hyperglycemia substantially reduces the risk of diabetes-related complications, including cardiovascular morbidity and mortality, reinforcing the public health significance of such screening approaches.^(55,56)^

A key limitation of this study is the relatively small sample size (n=21), which restricts the statistical robustness of the analyses and increases the risk of overfitting, particularly in the multinomial logistic regression and ROC curve models. Although these approaches were included to explore potential associations and diagnostic performance, their results should be interpreted with caution. In contrast, large-scale population-based studies, such as that of Montero et al^(41)^ provide greater reliability and control for confounding factors. Moreover, the small sample size increases the risk of overfitting, potentially resulting in unstable estimates and wide confidence intervals, limiting the precision and generalizability of the identified predictors. The present study is more appropriately framed as a pilot investigation, providing preliminary evidence on the feasibility and potential utility of the FINDRISC questionnaire in a dental setting. In addition, the use of a convenience sample from a dental school clinic may have introduced some bias, as such settings typically attract patients with more advanced treatment needs. This is reflected in the high proportion of individuals with severe disease in our cohort, with 71.4% presenting Stage IV periodontitis. Consequently, the findings may not be generalizable to populations with milder forms of periodontal disease or to community-based settings. Future studies should include larger and more diverse samples to improve external validity, allow for more robust multivariable modeling, refine cutoff points, and confirm the diagnostic performance of FINDRISC across different clinical contexts. Larger studies are needed to validate these associations and provide more robust estimates.

Another caveat of the present study relates to the diagnostic profile of the FINDRISC questionnaire. While the tool demonstrated high sensitivity (92-100%) and NPV (80-100%), supporting its use as a screening instrument to rule out dysglycemia, its relatively low specificity (26-44%) and PPV for T2DM alone (13%) indicate a substantial rate of false-positive results. This suggests that, although FINDRISC is effective in identifying individuals unlikely to have the disease, it has limited ability to confirm dysglycemia when used in isolation.

From a clinical perspective, its high NPV indicates that individuals with low FINDRISC scores are unlikely to have undiagnosed dysglycemia, reinforcing its role as an effective rule-out tool in dental settings. In this context, its primary utility lies in identifying low-risk patients, thereby supporting clinical decision-making and potentially reducing unnecessary referrals and laboratory testing. This trade-off is consistent with screening strategies, where maximizing sensitivity is prioritized to avoid missed diagnoses. Nevertheless, FINDRISC should be used as an initial risk stratification method and complemented by biochemical assessment, such as HbA1c measurement. Future studies should explore strategies to improve its specificity, including combining FINDRISC with clinical or biomarker-based approaches in dental settings.

## CONCLUSION

The combination of the FINDRISC questionnaire with clinical diagnosis of periodontitis suggests potential utility in identifying individuals with undiagnosed prediabetes or *diabetes mellitus* type 2. This highlights the pivotal role of dental professionals in systemic disease detection and management. The integration of simple, non-invasive screening tools into routine dental care may contribute meaningfully to the early identification and management of hyperglycemia, ultimately improving both oral and systemic health outcomes.

## Data Availability

The underlying content is contained within the manuscript.
